# Homœopathy: An Examination of Its Doctrines and Evidences

**Published:** 1852-04

**Authors:** 


Art. VII.—*Homœopathy:* An Examination of its Doctrines and Evidences. By Worthington Hooker, M. D., Author of "Physician and Patient," and "Medical Delusions." New York: Scribner, 145 Nassau street, 1851.

This work took the prize of the Medical Society of Rhode Island, as the best dissertation submitted on the claims of Homœopathy. It is unquestionably the most thorough, able and convincing work that has yet been called forth in the English language by that most absurd but captivating delusion. We venture to affirm, that no unprejudiced person can read this essay of Dr. Hooker's without feeling fully convinced of the absurdity of Homœopathy. The argument is overwhelming. It can not be answered, and we predict that no homœopathist will ever attempt to answer it. Cavil at it he may; but a reply in the shape of argument is out of the question.

We are aware that few of our readers are likely to be troubled with this form of empiricism, which is a plant that thrives best in the atmosphere of cities; but we can not withhold from them some passages with which we were particularly struck in this admirable little work.

We present them, first, with an incident in the life of the founder of homœopathy which has not often been alluded to, and which his admirers especially are not likely to mention. It is this:

In the early part of his career he appeared before the public as the seller of secret nostrums. "About the year 1800," says Dr. Leo Wolf, "Hahnemann advertised a new salt, of which he claimed the discovery, and which he sold at the modest price of a *louis d'or* per ounce. The Society for the Promotion of Natural Sciences, desirous of becoming acquainted with this new substance, had it analyzed by some of the most experienced chemists, who pronounced it to be nothing but common borax. He shortly afterward advertised an infallible preventive of scarlet fever; but being disappointed in its sale, he afterward confessed it to be nothing but a few grains of extract of belladonna dissolved in water." These transactions brand "the sage of Coethen" not only as a mercenary quack, but as a dishonest one.

Has any one among our readers ever attempted to compute the size of one of the infinitesimal doses? Dr. Hooker offers the following:

Let us try to get some idea of the minuteness of this dilution. Let us see what quantities of liquid would be required for the successive dilutions, if, instead of throwing away ninety-nine parts out of every hundred, the whole is retained. For the first dilution one hundred drops of alcohol would be used. For the second it would take ten thousand drops, or about a pint. For the third it would take one hundred pints. For the fourth ten thousand pints. And now it mounts up rapidly at each dilution. For the ninth dilution it would take ten billion of gallons, which, according to the computation of Dr. Panvani, equals the quantity of water in the Lake Agnano, which is two miles in circumference. For the twelfth dilution a million of such lakes would be required, or as it is reckoned by Dr. Post of New York, (from whom I shall take the liberty to borrow the remaining calculations rather than attempt them myself,) it would require five hundred lakes as large as Lake Superior. The fifteenth dilution would require a quantity of alcohol greater in bulk than the earth. The eighteenth would require a quantity greater than the volume of the sun. And the thirtieth, the one which Hahnemann insists upon as being the best for common use, would take a quantity of alcohol exceeding the volume of a quadrillion of suns.

Upon the well known principle of Hahnemann, Dr. Hooker makes the following conclusive remarks:

If *similia similibus curantur* be the *sole* law under which cures are effected, then we should be able to prove, either that the vital powers are never competent to cure disease alone and unassisted by remedies, or, that they do it in conformity with the Homœopathic law. Hahnemann accepts the first horn of the dilemma; and expressly asserts that the cures alleged to be effected by the *vis medicatrix naturæ* are not cures. He has but a poor opinion of the efforts of the "unintelligent vital powers," and quarrels with "vulgar practice" for its imitation of nature's bungling operations.

Now there is no fact more thoroughly established, both by common and professional observation, than that the curative tendency in the system is competent to cure a large proportion of the attacks of disease without the assistance of any remedy. This is certainly true of the numberless trivial ailments, slight colds, temporary headaches, etc., which so often get well without medicine, and alike with or without its shadow, the Homœopathic globule. Perhaps, however, the Homœopathist would claim that these are not really diseases, although each case maniestly presents its group, its "totality of symptoms." I remark again, then, that what I have said of the curative tendency of nature is certainly true of all mild cases of what are termed self-limited diseases—those which have a certain defined set of processes to go through, such as measles, small-pox, etc. When these maladies have finished their course, the vital powers restore the healthy condition of the system, removing all the consequences of the disease. The same is true too of other diseases. In all mild cases, with proper diet and regimen, the vital powers are able to cure them. And in the practice of every judicious physician, a large share of the medication employed aims at assisting the curative tendency of nature, and removing obstacles out of its way, so that its action may be free and undisturbed.

As then the *vis medicatrix naturæ* effects cures, it has its principles upon which it does this—in other words, it has its laws of cure. The Homœopathic law of cure then is not the *sole* law.

Homœopathy has its statistics, and professes by these to prove that it is more successful than the regular practice. Dr. H. thus dismisses this pretension:

If the statistics of Homœopathy be tested by the principles which I have indicated, they will be found wanting in those qualities which command our confidence. We will take for example its statistics of cholera. It was stated, after the first visitation of this disease in Europe, as the grand result of these statistics, that while the average mortality under the "regular" treatment was about forty-nine in one hundred, under Homœopathic treatment it was only about six in one hundred. This, you will observe, is an enormous difference. If the statement was really true, it is wonderful that the Homœopathic treatment of this disease has not been adopted by this time all over the world. *It would have been, if the statement had been believed.* But it has not been believed.

It is a significant fact as bearing upon the pretensions of Homœopathy, that while it has gained with the populace, it has been steadily rejected by the great body of the profession. On this point Dr. Hooker has the following forcible thoughts;

Homœopathy has been fairly before medical men for fifty years; and the profession has passed its verdict upon it in the most deliberate and positive manner. Some are disposed to think that this verdict is good for nothing, and openly charge medical men, as a body, with a wilful blindness to the truth of Homœopathy. If this charge be well founded, the medical profession are governed in relation to this doctrine by a spirit altogether different from that which they have manifested towards all other new doctrines and opinions. Look over the whole history of medicine, and observe the course which the profession have pursued in regard to the numberless doctrines and theories which have arisen from time to time. As they have passed away one after another, they have been examined and sifted by medical men, and while much has been rejected, much has been retained and added to the permanent treasures of our science. And you can not adduce a single instance, in which anything that time has shown to be valuable, has not in a very short period gained a strong hold upon the professional mind, however great might be the opposition to it at its first promulgation. If Homœopathy be truly valuable, it is the first thing of this character which has failed to be thus established among medical men. It is a single solitary exception, showing an irrational obstinacy which the profession have certainly not been wont to manifest.

It is a common assertion of the Homœopathists, as of the advocates of other quackeries, that the profession at first and for a long time rejected the discoveries of Harvey and Jenner; but the statement, as every physician who is acquainted with the history of medicine well knows, is false.

Dr. Hooker continues :

Let us suppose, now, a parallel case. Suppose that fifty years ago some theologian had broached a new mode of biblical interpretation, which, if true, would set aside all old rules and modes, as Hahnemann's system, if true, would do in medicine—that, though the author of this system was a talented man, few among all the regularly educated divines had adopted it—that of this number but a very few were men of any standing—that the great majority of those who proclaimed the new doctrine were poorly educated men, and that this new sect opposed themselves to all "regular" theologians of every name, and set up schools to supply the community with divines, who are educated in nothing but the absurdities of their system. Would it not, I ask, be claimed of us laymen, that we should believe, almost as a matter of course from the very reception thus given to the new doctrine by theologians, that it was false? Would it not be said, that it is not to be supposed theologians, with all their various differences, would unite as a body in rejecting what is truly valuable; and that if the doctrine had any truth in it, it could certainly get a lodgment in some of all the various theological schools, and that schools need not therefore be instituted purposely for its propagation?

The parallel is complete in this case. It is not defective in a single particular; and yet if we should assert that the rejection of this new mode of interpretation for fifty years by theologians as a body is no evidence against its truth, it would be taking the same ground that many clergymen take in relation to the rejection on the part of physicians of Hahnemann's mode of interpreting disease and its cure.

The following parallel between Homœopathy and the systems of empiricism which share with it the delusion of the popular mind, will strike every one as forcible and just.

Homœopathy and its sister delusion, Thompsonianism, strongly resemble each other in the manner in which they prosecute their claims. Though they move in different spheres, their tactics are very much the same. Though Homœopathy may look with contempt upon the coarse radicalism of her vulgar and ignorant sister, she has the same radicalism in a more refined and specious form. Both cry out against the "regular" profession; and the tendency of the efforts of both, however stoutly the genteel and learned patrons of Homœopathy may deny it, is to destroy the safeguards which secure to the community a well-educated body of medical men. Other systems, as Chrono-Thermalism, Eclecticism, etc., have also arisen, and have taken the fashion of their measures from Hahnemannism and Thompsonism, and have joined with them in the great work of medical radicalism.

